# A randomized trial to determine the minimum effective lidocaine volume for median nerve block using hydrodissection

**DOI:** 10.1038/s41598-021-03660-7

**Published:** 2022-01-07

**Authors:** Eric Dufour, Souhail Jaziri, Marie Alice Novillo, Lila Aubert, Anne Chambon, Rainer Kutz, Alexandre Vallée, Marc Fischler

**Affiliations:** 1 Department of Anesthesiology, Centre Hospitalier de la Côte Basque, 64109 Bayonne, France; 2 Department of Anesthesiology, Hopital Privé de l’Ouest Parisien, 78190 Trappes, France; 3 Department of Pharmacy, Centre Hospitalier de la Côte Basque, 64109 Bayonne, France; 4 Department of Clinical Research and Innovation, Hôpital Foch, 92150 Suresnes, France; 5 Department of Anesthesiology, Hôpital Foch, 40 rue Worth, 92150 Suresnes, France

**Keywords:** Phase IV trials, Pharmacodynamics

## Abstract

Ultrasound-guided hydrodissection with 5% dextrose in water (DW5) creates a peri-nervous compartment, separating the nerve from the neighboring anatomical structures. The aim of this randomized study was to determine the minimum volume of lidocaine 2% with epinephrine 1:200,000 required when using this technique to achieve an effective median nerve block at the elbow in 95% of patients (MEAV95). Fifty-two patients scheduled for elective hand surgery received an ultrasound-guided circumferential perineural injection of 4 ml DW5 and an injection of local anesthetic (LA) following a biased coin up-and-down sequential allocation method. A successful block was defined as a light touch completely suppressed on the two distal phalanges of the index finger within a 30-min evaluation period. The MEAV95 of lidocaine 2% with epinephrine was 4 ml [IQR 3.5–4.0]. Successful median nerve block was obtained in 38 cases (82.6%) with median onset time of 20.0 [10.0–21.2] minutes (95% CI 15–20). The analgesia duration was 248 [208–286] minutes (95% CI 222–276). Using an ultrasound-guided hydrodissection technique with DW5, the MEAV95 to block the median nerve at the elbow with 2% lidocaine with epinephrine was 4 ml [IQR 3.5–4.0]. This volume is close to that usually recommended in clinical practice.

*Trial registration* clinicaltrials.gov. NCT02438657, Date of registration: May 8, 2015.

## Introduction

Although rare and despite the availability of ultrasound imaging, local anesthetic (LA) systemic toxicity, a life-threatening complication partly related to the administered dose, still occurs^1^. Hence, using the lowest effective LA dose required to achieve an adequate surgical block is one of the key prevention strategies for such complications^2^.

Only a few studies have evaluated the volume-response relationship of LAs for peripheral nerve blocks^3–5^. Furthermore, the reproducibility of these studies for everyday clinical practice remains in question, suggesting that the manipulation of small aliquots and further needle repositioning for consecutive injections are not straightforward and require a high level of expertise to interpret a correct distribution around the nerve and detect an intraneural injection^4–6^. The latter is important since it has been clearly reported that intraneural injections, whether intended or unintended, significantly reduce the LA dose and improve the success rate to achieve an effective block^7–9^. With the hydrodissection technique^10^, an injection of 5% dextrose in water (DW5) can mechanically separate the nerve from its hyperechoic surrounding fascial layers, aiming to standardize a perineural compartment into which the active drug, i.e., the LA, is injected. As an increase in cross-sectional area (CSA) is a clue for intraneural injection, we considered an extraneural compartment to be created when the needle passed through multiple sequential layers and the nerve did not swell during DW5 injection. The present study aimed to evaluate, using a hydrodissection technique with DW5, the minimum volume of lidocaine 2% with epinephrine 1:200,000 required for an effective ultrasound-guided median nerve block at the elbow in 95% of patients (MEAV95).

## Methods

This prospective randomized double-blinded study was institutionally sponsored. It was conducted at two health institutions in France (one non-profit and one for-profit). This study was approved by an Ethics Committee (Comité de Protection des Personnes Sud-Ouest et Outre-Mer III, 07/05/2014, Pr Cuny Chairperson; IRB 2014-000571-50) and written informed consent was obtained from all subjects participating in the trial. The trial was registered prior to patient enrolment at clinicaltrials.gov (NCT02438657, Date of registration: 08/05/2015). This manuscript adheres to the applicable Consolidated Standards of Reporting Trials (CONSORT) guidelines.

### Participants

The inclusion criteria were American Society of Anesthesiologists physical status I–II, age 18–80 years, and scheduled elective ambulatory surgery of the hand involving the median nerve lasting less than 30 min and performed with an ultrasound-guided nerve block at the elbow.

The exclusion criteria were an inability to speak French, pregnancy or lactation, diabetes mellitus, or bleeding, contraindication to regional anesthesia (infection at the puncture site notably) or lidocaine or epinephrine (allergy, porphyria, severe cardiomyopathy, epilepsy, hyperthyroidism), and preexisting central or peripheral neuromuscular disease (including carpal tunnel syndrome). A sensory and motor examination of the median nerve of each potential participant was then performed to exclude those with abnormalities.

Patients were excluded from the study if they received an intraneural injection into the median nerve during the hydrodissection procedure.

### Randomization and allocation

Each patient received a unique patient number and a randomization number (patient code). For each subsequent patient, the anesthesiologist connected to an Interactive Web Response System managed by an independent Contract Research Organization (https://www.ab-plus.com/) using a protected password just before the nerve block. This system gives the result of the preceding patient (success or failure based on the primary criterion) and provides the volume to be injected from a predefined list established before the beginning of the protocol. The software used to allocate the patients into groups was also used to collect data from the investigator in real-time through an electronic report form, ensuring concealment.

### Study protocol

After contacting the randomization center, a nurse prepared an unlabeled syringe containing the volume of 2% lidocaine with epinephrine for the median nerve block. A first anesthesiologist performed the block, and a second anesthesiologist evaluated the sensory nerve block. Thus, both anesthesiologists, like the surgeon and the patient, were blinded to the volume administered, except for the starting dose which was administered for the first patient (2 ml). At the end of the evaluation period, a second anesthesiologist informed the nurse of the success or failure of the block allowing the randomization of the next patient.

In the preoperative room, patients were as usual with electrocardiogram, noninvasive arterial blood pressure, and pulse oximetry. A 20-gauge IV catheter was placed on the hand contralateral to the operative site. No sedation was given. Nerve blocks were performed by E.D. or J.S using a LOGIQe® (GE Healthcare; Buc, France) or a Mindray TE7 (Mindray Medical; Créteil, France), respectively. After sterilizing the skin with iodine polyvidone, the ulnar, radial, and median nerves were successively blocked 4 to 5 cm above the elbow using three different puncture sites. A transverse view of each nerve was obtained with a high-frequency linear probe protected by a sterile sheath cover: proximal to the medial epicondyle for the ulnar nerve at the medial aspect of the abducted and externally rotated arm; proximal to the lateral epicondyle for the radial nerve at the lateral aspect of the arm internally rotated; and at the medial aspect of the arm abducted at right angles to the body for the median nerve.

After skin infiltration with plain lidocaine 1%, a 22-gauge, 50-mm-long insulated needle with a 30° bevel (Sonoplex®, Gamida; Trappes, France or Echoplex®, Vygon; Écouen, France) was advanced using a short-axis in-plane approach and placed adjacent to the targeted nerve, avoiding injection through the epineurium. For the ulnar and radial nerves, the LA solution of 4 ml of 1.5% lidocaine with epinephrine 1:200,000, was injected gradually after aspiration tests, and the needle tip was redirected until an entire circumferential spread was obtained. The median nerve block was performed using a hydrodissection technique with a syringe containing 4 ml of D5W: small aliquots were gradually injected, and the needle was redirected as needed to mechanically peel off the hyperechoic sheath surrounding the median nerve and achieve a circumferential hypoechoic space using a static axial ultrasound view. If several layers were visible around the nerve, an effort was made to pass through the innermost layer without crossing the presumed epineural sheath. The injection was considered extraneural in the absence of real-time nerve swelling. The needle was held in this space with a static position, and an unlabeled syringe containing the randomized volume of lidocaine 2% with epinephrine 1:200,000 was slowly injected. If real-time nerve swelling was observed during the hydrodissection procedure, we considered that the injection was subepineural, and the needle was immediately removed and repositioned outside the epineural sheath. Then, 4 ml of lidocaine 2% with epinephrine was injected, and the patient was excluded from analysis.

Any potential surgical area not completely anesthetized after a 45-min evaluation was supplemented at the elbow. In the case of pain during surgery, an additional infiltration of plain lidocaine 1% was performed at the surgical wound by the surgeon.

During surgery, propofol was injected if the patient experienced anxiety, and sufentanil was administered if tourniquet pain occurred.

Postoperative analgesia was provided with a combination of paracetamol 1 g, ketoprofen 50 mg, and tramadol 50 mg every 6 h depending on pain intensity.

Before discharge, patients were asked to note the time at which sensory and motor function of the first three fingers returned to normal and the time when pain was first felt. Patients were contacted by phone the next day to report the block duration, analgesia duration, and level of satisfaction.

Postoperative routine neurologic evaluations (including evaluations of pain, anesthesia, paresthesia, and weakness of the operated limb) were performed on postoperative day 3 in the outpatient clinic and 1 month postoperatively by the surgeon and via a phone survey by the anaesthesiologist.

### Data collection

The evaluation of sensory and motor function after the median nerve block was performed using cold and light touch tests and the index flexion test.

Sensory block was evaluated through a contralateral comparison of cold sensation elicited by ice and light touch sensation performed on the palmar aspect of the two distal phalanges of the index finger and on the thenar eminence. The sensory rating was quantified as follows for each test: normal sensation = 0, reduced sensation = 1, and total loss of sensation = 2. The motor examination was performed by asking the patient to flex the two distal index phalanges and scored as follows: no loss of strength = 0; reduced strength in comparison with the contralateral index finger = 1; and total immobility = 2.

Sensory and motor assessments were performed every 5 min for 30 min after needle removal and at 45 min in case of median nerve block failure (i.e., index light touch score 0 or 1 at 30 min).

Before discharge, patients were asked to note in a written document the time at which sensory and motor function of the first three fingers returned to normal and the time they felt pain and were told that they would be telephoned the next day.

Block duration was defined as the interval between when the anesthesiologist finished performing the block and time to sensory and motor recovery. Analgesia duration was defined as the interval between when the anesthesiologist finished performing the block and when pain returned. The level of patient satisfaction regarding anesthesia was also evaluated using an 11-point scale (0 = not at all satisfied; 10 = extremely satisfied) by phone.

### Study outcomes

The primary outcome was the volume of lidocaine 2% with epinephrine required to achieve a median nerve block at the elbow in 95% of patients. A successful block was defined as a light touch completely suppressed (= score of 2) on the palmar aspect of the two distal phalanges of the index finger within the 30-min evaluation period.

The secondary outcomes included block procedure time, success rate of the median nerve block at 30 min and 45 min, nerve block onset time, additional nerve block and surgeon infiltration, median nerve block and analgesia duration, patient satisfaction, and neurologic complications.

### Statistical analysis

The EC95 that determined the volume of lidocaine 2% with epinephrine which was sufficient to achieve a successful axillary block was estimated using the biased coin up-and-down design sequential method (BCD)^11,12^.

The BCD is flexible and can target any prespecified toxicity rate^11^. The BCD is based on the theory of random walk and assigns patients to a dose level one at a time, i.e., *s* = 1. Suppose that the *i*th patient is treated at dose level *j*. To determine a dose for the next patient,

- if *Y*_*i*_ = 1, we deescalate the dose level to *j − *1;
- if *Y*_*i*_ = 0, we escalate the dose level to *j* + 1 with a probability of *ϕ/*(1 − *ϕ*), otherwise we retain the current dose level *j*.

The BCD method is based on three simple principles:

- the desired dose must be within the tested interval, it cannot be calculated by extrapolation.
- taking into account the fact that it is an ED95 which is sought, a failure must seek immediately a higher dose.
- for the same reasons, only prolonged series of successes should lead to a reduction in the dose. The higher the desired efficiency percentage, the longer the series.

The initial dose chosen by the investigator was 2 ml, which was derived from a previous study that showed that 1 ml per nerve of lidocaine 2% with epinephrine was sufficient to achieve a successful axillary block^4^. If the anesthetic effect achieved 30 min after the realization of the nerve block allows the intervention to proceed, the block is considered a success; otherwise, it is a failure. In case of failure, an increase of 0.5 ml to the dose that led to this failure (Vn − 1) was used for the next patient (Vn). If successful, the algorithm of randomization determined whether the dose administered to the previous patient (Vn − 1) should be retained or if it should be reduced by 0.5 ml for the next patient (Vn). Randomization was designed so that a dose reduction was assigned only if enough successes were recorded with the previously used dose. This number was calculated based on the total sample size calculated, in such a way that it guarantees cases of dose reduction and that the previous dose led to a sufficient percentage of successes (in our case, 95%). If Vn was the last dose administered and the alpha risk was 0.05 (its complement to 1 was therefore 0.95), i the increment of the DE95, and s the number of successes with Vn, thus the most exact DE95 was calculated by the formula: Vn + i × (1 − 0.95s). We considered that 1 ml was the minimal administrable volume, and 4 ml was the maximal volume administered.

Assuming a fixed-sample biased coin design and a fixed minimal number of successes for different dose distributions, sample sizes, and number of successes, stabilization of the estimated parameters occurred after the enrollment of a minimum of 45 subjects according to statistical references^11,12^, even when applying the assumption that the probability of receiving a lesser volume after a successful response is equal to a theoretical value of 0.053. The EC95 was estimated by calculating a modified isotonic estimator (MIE)^12^. To estimate the MEAV95, a minimum of 11 positive responses was required depending on the experience of the investigators, with a two-sided significance level of 5% and an increment of 0.5 ml. One-sample t-tests were performed. The R 2.14.1 program (R foundation for Statistical Computing, Vienna, Austria) was used for this calculation. The 95% confidence interval (CI) was obtained using a parametric bootstrap routine and calculated by a bias corrected percentile method^11^.

The data are presented as numbers (percentage) and median [25th–75th percentiles] (95% confidence interval of the median). The MEAV95 is presented as the median [25th-75th percentiles]. The Spearman correlation test was used to determine the relationship between the volume of LA administered and block onset time.

### Ethical approval

All procedures performed in studies involving human participants were in accordance with the ethical standards of the institutional and with the 1964 Helsinki declaration and its later amendments or comparable ethical standards.

### Ethics committee approval

This study was approved by an Ethics Committee (Comité de Protection des Personnes Sud-Ouest et Outre-Mer III, May 7, 2014, Pr Cuny Chairperson; IRB 2014-000571-50).

### Informed consent

Informed consent was obtained from all individual participants included in the study.

## Results

**Figure 1:**
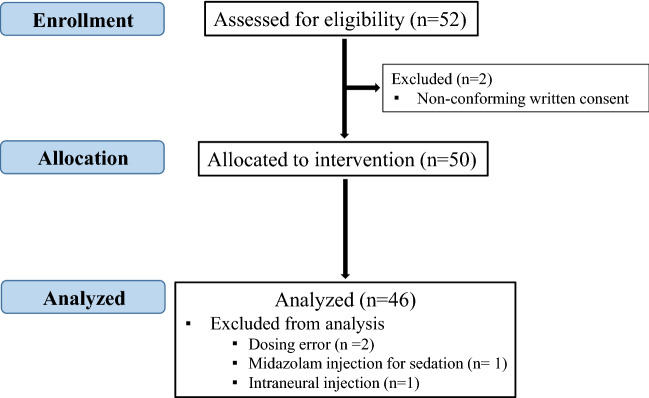
Flow chart.

Fifty-two patients were enrolled in the two centers from May 2015 to October 2018, the first 17 patients were from center 1, and the others were from center 2. Six patients were excluded for the following reasons: incomplete consent (two patients), protocol deviation (3 patients) and intraneural injection (one patient). Consequently, 46 patients were analyzed (Fig. 1).

The demographic data and surgical procedures are summarized in Table 1.

**Table 1:** Demographic data and surgical procedures.

	N = 46
Male/female	23 (50)/23 (50)
Age	49 [31–61] (40– 55)
Height	169 [163–176] (166–172)
Weight	72 [62–83] (66–80)
ASA score 1/2	29 (63)/17 (37)
**Surgical procedure**	
Finger and hand wounds	20 (43.5)
Synovial cyst	3 (6.5)
Trigger finger	14 (30.4)
Whitlow	6 (13.0)
Other	3 (6.5)

The results are presented as numbers (percentages) and medians [25th–75th percentiles] (95% confidence intervals).

The median nerve block procedure time was 150 [104–202] sec (112–180).

**Figure 2:**
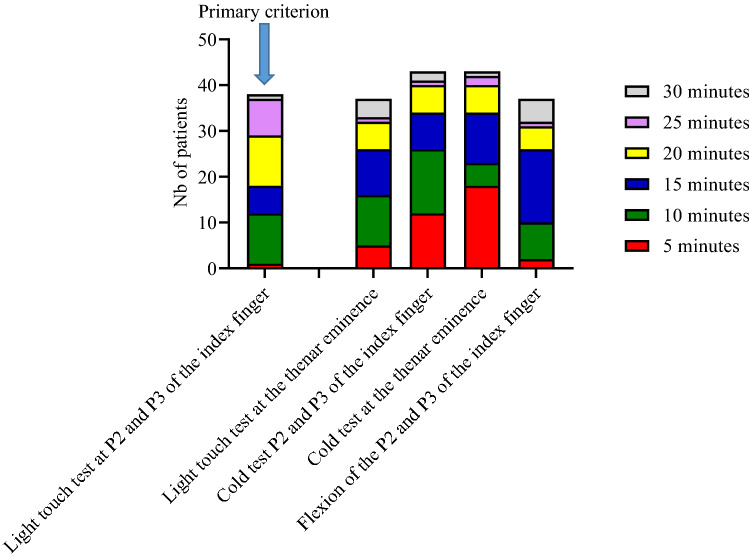
Repartition of onset time for successful block according to the sensory and motor test results.

A successful median nerve block within 30 min, our primary criterion, was obtained in 38 cases (82.6%) with an onset time of 20.0 [10.0–21.2] minutes (95% CI 15–20). The reported onset times of a complete block according to the sensory and motor tests used were highly variable (Fig. 2).

**Figure 3:**
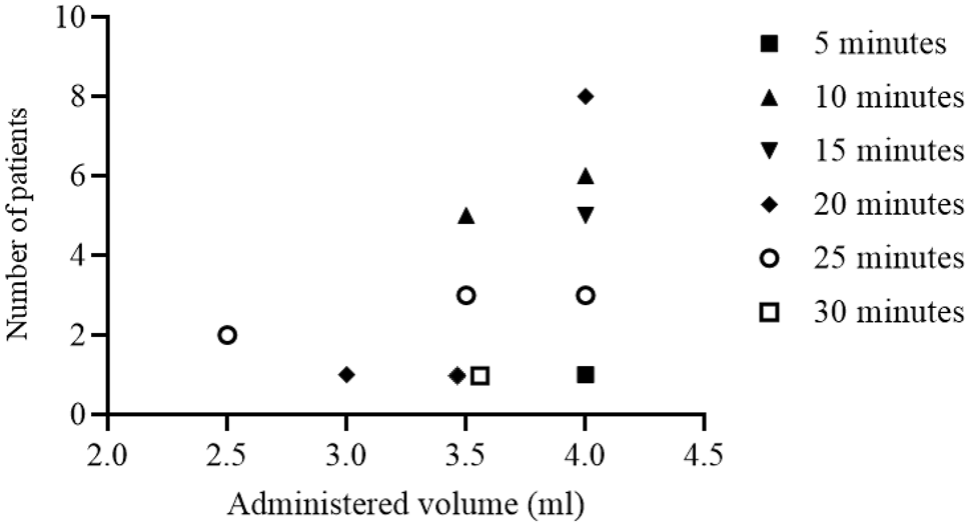
Relation between LA volume and block onset time for successful blocks. A successful block was defined by a score = 2 with a light touch felt on the P2 or P3 of the index finger.

The relationship between the volume of LA administered and block onset time was highly variable regardless of the administered volume (*p* = 0.077; Fig. 3).

At the 45-min evaluation, an additional two patients achieved a complete median nerve block, increasing the success rate to 86.9%. Among the 46 patients, 44 patients (95.6%) underwent surgery without the need for a rescue block or additional infiltration.

**Figure 4:**
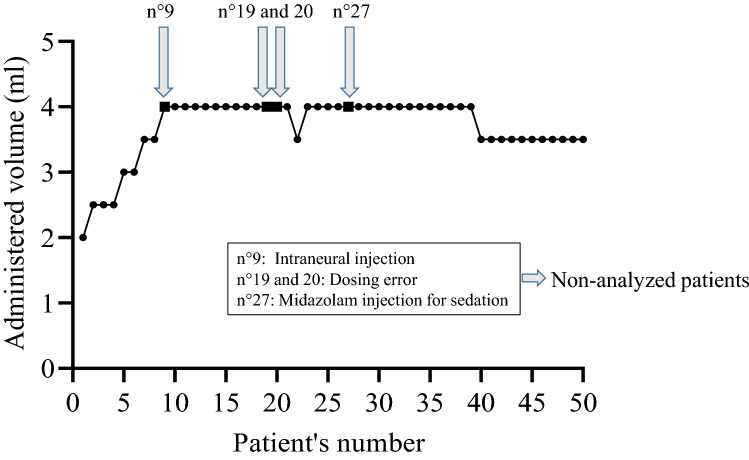
Up-and-down evolution for median nerve block in patients receiving an ultrasound-guided median nerve block using hydrodissection with DW5 and 2% lidocaine with epinephrine.

The minimum effective volume of lidocaine 2% with epinephrine required to achieve a complete median nerve block in 95% of patients (MEAV95) calculated using the biased coin up-and-down design was 4 ml [25th–75th percentiles: 3.5–4.0] (Fig. 4).

The block duration time was 320 [255–390] minutes (95% CI 281–348). The analgesia duration was 248 [208–286] minutes (95% CI 222–276).

Paresthesia was observed for five patients. No vascular puncture was observed.

In total, 78% of patients had a satisfaction score > 8, and 54% had a score = 10.

Routine neurologic examinations performed by the surgeon and anesthesiologist did not detect any complications.

## Discussion

Our study establishes that the MEAV95 of lidocaine 2% with epinephrine required to achieve a median nerve block at the elbow with a hydrodissection technique with DW5 is 4 ml [interquartile range 3.5–4.0].

Although using the lowest effective dose is one of the recommendations for preventing LAST, using a too low dose may be inappropriate in daily practice considering the high level of expertise required to manipulate and distribute a small aliquot of LA around the nerve with ultrasound guidance^4–6^. The entire LA injected volume during nerve block procedure cannot be involved in the efficacy study if its distribution, observed after the injection does not appear to be in the perinervous compartment. Furthermore, manipulation of a very low volume of LA increases the risk of an intraneural injection, with finding reported to be difficult for a volume of 0.5 ml^13^. This is problematic when modeling the volume-effectiveness relationship^4,5,14^.

We tried to reduce this bias by using a hydrodissection technique with DW5 to create a standardized extraneural pharmacologic compartment and evaluate a dose–effect relationship.

Hydrodissection corresponds to an opening or space between anatomical structures and is different from hydrolocalization, which also involves an injection of fluid such as 5% dextrose but to confirm needle tip position^15^. Thus, we first determined the optimal perinervous conferential diffusion compartment with an injection of 4 ml DW5 solution and subsequently injected the LA studied dose.

We thought that a 4 ml volume of DW5 could facilitate the procedure, as this volume is usually used in daily practice. We also used an ultrasound imaging-based definition, i.e., “circumferential perinervous non-swelling compartment,” rather than a histological definition like “subparaneural injection”^16^, the interpretation of which remains controversial because of insufficient ultrasound resolution to identify the paraneurium^17^. Hydrodissection with DW5 can theoretically reduce the risk of nerve toxicity related to intraneural injections of LA since once noted, the needle can be removed from the epineurium before the injection of LA.

Finally, we used a model of a unique nerve to prevent an unwanted diffusion spread of the LA to the neighboring nerves that occurs at the axillary approach^4,14^, and our primary endpoint was to determine the volume for a 95% success rate that was relevant for clinical practice.

Previous studies have evaluated the efficacy of low volumes for ultrasound-guided median nerve block and found doses ranging from 1 to 3.1 ml depending on the LA studied, the volume injected relative to the nerve cross-sectional area or the anatomical approach used^4,5,14^.

Thus, comparisons with these studies are difficult. Our reported MEAV95 of lidocaine 2% is close to the usual volume used in daily practice^18^. The success rate for median nerve block was 82.6%, and 95.6% of patients underwent surgery without supplemental LA injection, suggesting that our technique is effective.

We cannot exclude the impact of dilution of the LA by DW5 during the hydrodissection procedure, although in a previous study, we reported that creating a circumferential perineural area of spread by hydrodissection with D5W prior to LA administration does not hamper nerve block efficacy^10^. Another explanation could be the abundance of non-neural tissue at the elbow in contrast to that encountered through a more proximal axillary approach, which requires a larger efficacy dose to reach the neural tissue^19^.

There was great variability in onset time according to the various outcomes. In the same way, modifying the time threshold to define block success, which was arbitrarily set at 30 min in our study, could have a direct influence on the results. Furthermore, we observed that the onset time also varies for patients receiving the same volume of lidocaine. No correlation between volume injected and onset time has previously been observed^5^. Considering that onset time reflects the difficulty of the LA reaching the neural structures, we believe that different perinervous compartments may exist despite the observation of similar “non-swelling nerve” ultrasound images and that the number of layers through which the LA has to diffuse through explains the variability in onset time to obtain an effective nerve block^20,21^. These observations raise the question of result interpretation and standardization in an up-and-down pharmacodynamic protocol model.

We used lidocaine since a short block duration with lidocaine allows for rapid recovery, which is advantageous for ambulatory surgery but requires prevention of pain reappearance. Consequently, our results are not applicable to other types of LAs or to other nerves.

### Strength and limitations

This study was double-blinded, and we chose to use the biased coin up-down sequential method and not the Dixon up-and-down method, which requires a logistic or a probit regression to extrapolate to higher quantiles (e.g., MEV95)^22^. We used the biased coin design, which directly gives MEAV95 for ultrasound-guided peripheral nerve blocks^23,24^. However, our study suffers from several limitations.

The study was performed in two centers successively, which raises the problem of reproducibility. However, the two anesthesiologists who performed the blocks applied the same technique, and effort was made to standardize the procedure with an accurate ultrasound definition of the extraneural compartment into which the LA was injected. Finally, all investigators attended a meeting about the trial before the inclusion of the first patient.

Although real-time nerve swelling has been reported to be reliable for detecting low-volume intraneural injection^25^, we did not specifically examine other signs of intraneural injection, such as indentation of the nerve wall by the needle or changes in echogenicity during injection^13^; additionally, we did not use injection pressure monitoring to aid in the identification of intraneural puncture. This is of particular interest since intraneural injection provided a faster onset and better success rate than subparaneural injection and obviously influenced the dose–effect relationship^8^.

While perineural injections of DW5 do not lead to long-term neurologic sequelae in humans or animals^26^, the consequences of intraneural injection of DW5 have not been studied, to the best of our knowledge.

Finally, source documentation verification (SDV) was performed after the last patient’s inclusion, which led to the withdrawal of six patients from the analysis. We did not consider this problem when we designed the study, but these six patients were treated after the excluded patients and thus had their dose defined based on the results of the excluded patients. In practice, as shown in Fig. 4, our results were not affected, but we feel it is important to stress the importance of verifying each patient's data before including the next patient when using Dixon's method.

## Conclusion

In conclusion**,** our study demonstrated that the MEAV95 to block the median nerve at the elbow is close to the dose usually used in clinical practice. Further studies using high-definition ultrasound imaging could help to identify the perineural diffusion space, thus enabling pharmacodynamic studies. The interest of applying the hydrodissection method to facilitate the perinervous administration of very small doses of LA in daily practice remains to be confirmed.

## Data Availability

Data are available upon reasonable request to the corresponding author.
